# Pioneering Robotic Liver Surgery in Germany: First Experiences with Liver Malignancies

**DOI:** 10.3389/fsurg.2015.00018

**Published:** 2015-05-20

**Authors:** Roland S. Croner, Aristotelis Perrakis, Maximillian Brunner, Klaus E. Matzel, Werner Hohenberger

**Affiliations:** ^1^Department of Surgery and Liver Center, University Hospital Erlangen, Erlangen, Germany

**Keywords:** liver resection, minimally invasive, robotic, liver malignancy, HCC, colorectal cancer

## Abstract

**Background:**

Minimally invasive liver surgery is growing worldwide with obvious benefits for the treated patients. These procedures maybe improved by robotic techniques, which add several innovative features. In Germany, we were the first surgical department implementing robotic assisted minimally invasive liver resections.

**Material and methods:**

Between June 2013 and March 2015, we performed robotic based minimally invasive liver resections in nine patients with malignant liver disease. Five off these patients suffered from primary and four from secondary liver malignancies. We retrospectively analyzed the perioperative variables of these patients and the oncological follow up.

**Results:**

Mean age of the patients was 63 years (range 45–71). One patient suffered from intrahepatic cholangiocellular, four from hepatocellular carcinoma, and four patients from colorectal liver metastases. In six patients, left lateral liver resection, in two cases single segment resection, and in one case minimally invasive guided liver ablation were performed. Five patients underwent previous abdominal surgery. Mean operation time was 312 min (range 115–458 min). Mean weight of the liver specimens was 182 g (range 62–260 g) and mean estimated blood loss was 251 ml (range 10–650 ml). The mean tumor size was 4.4 cm (range 3.5–5.5 cm). In all cases, R0 status was confirmed with a mean margin of 0.6 cm (range 0.1–1.5 cm). One patient developed small bowel fistula on postoperative day 5, which could be treated conservatively. No patient died. Mean hospital stay of the patients was 6 days (range 3–10 days). During a mean follow up of 12 months (range 1–21 months), two patients developed tumor recurrence.

**Conclusion:**

Robotic-based liver surgery is feasible in patients with primary and secondary liver malignancies. To achieve perioperative parameters comparable to open settings, the learning curve must be passed. Minor liver resections are good candidates to start this technique. But the huge benefits of robotic-based liver resections should be expected in extended procedures beyond minor liver resections with the currently available technology.

## Introduction

Minimally invasive liver surgery is growing worldwide. Initially recommended for smaller benign lesions only, recent meta-analysis demonstrates the value for malignant tumors as well. No differences in the long-term survival and tumor recurrence for patients between open and laparoscopic procedures were observed. But the blood loss and postoperative complication rates seem to be lower in minimally invasive procedures (1, 2). Therefore, the laparoscopic left lateral resection was recently recommended as standard of care (3). Currently, techniques for the minimally invasive access to all liver segments for minor and major resections are described (4). Robotic-based surgical techniques are currently in the focus of interest and are evaluated for various indications. Even liver surgery was already performed by robotics, which may add several benefits to improve standard laparoscopic operations. The excellent three dimensional view, use of an endowrist, three surgeon controlled arms, which result in an excellent stable operation field enabling very precise maneuvers are arguments for robotic tools (5). Nevertheless, a recent international survey on technical aspects of liver surgery showed that Germany is worldwide behind in the performance of minimally invasive liver resections programs (6). We picked up this issue and started as the first center in Germany minimally invasive robotic-based liver resections. We are still in a learning curve, but the procedure and the set up are standardized. We share here our initial experiences in liver malignancies.

## Materials and Methods

### Patients and tumors

Between June 2013 and March 2015, nine patients with malignant liver tumors were operated with robotic-based minimally invasive surgery. One of these patients suffered from intrahepatic cholangiocellular carcinoma (CCC), four patients from hepatocellular carcinoma (HCC), and four patients from metachronous colorectal liver metastases (CRC). Only patients with ≤2 unilateral lesions identified by preoperative imaging techniques (CT-scan, MRI) were selected. The mean tumor size was 4.4 cm (range 3.5–5.5 cm). Two patients with CRC underwent previous laparoscopic rectum resection, one patient robotic-based rectum resection and one patient open rectum extirpation. One patient with HCC was treated with laparoscopic sigmoid resection for diverticulitis prior to liver resection. Patient demographics are listed in Table 1.

**Table 1:** **Demographic, perioperative, and histopathology data of patients, which underwent robotic-based liver resection for primary and secondary liver malignancies**.

Patient	Age (years)	Tumor	Tumor size (cm)	R-status	Surgery	Operation time (min)	Previous abdominal surgery	Liver fibrosis (Ishak-scoring)	Hepatic steatosis	Postop. morbidity	Discharge (postop. day)
1	71	CCC	5.5	0	Left lateral	458	None	n.d.	20–40%	None	7
2	45	CRC	3.5	0	Left lateral	368	Robotic sigmoid	0	None	None	6
3	75	CRC	4.5	0	Left lateral	314	Lap. rectum	0	5%	None	5
4	66	HCC	5	0	Left lateral, gallblader	405	Open sigmoid	2	5%	None	9
5	64	HCC	3.1	0	Segment III	138	None	n.d.	n.d.	None	6
6	58	HCC	5	0	Ablation segment II/III and IV	115	None	4–5	n.d.	None	3
7	69	CRC	4.1	0	Left lateral	228	Open rectum extirpation	1	5–10%	Small bowel fistula	10
8	62	HCC	5	0	Left lateral	403	None	6	20–30%	None	6
9	57	CRC	3.5	0	Segment V, gallblader	375	Lap. rectum	1	n.d.	None	6

*n.d., not determined*.

### Operative setting

**Figure 1:**
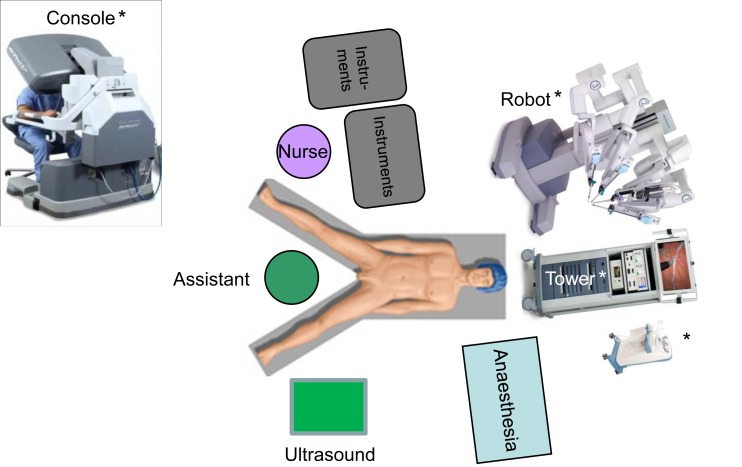
**Operative setting for robotic based liver surgery**. * © 2015 Intuitive Surgical, Inc. All other figures are original.

**Figure 2:**
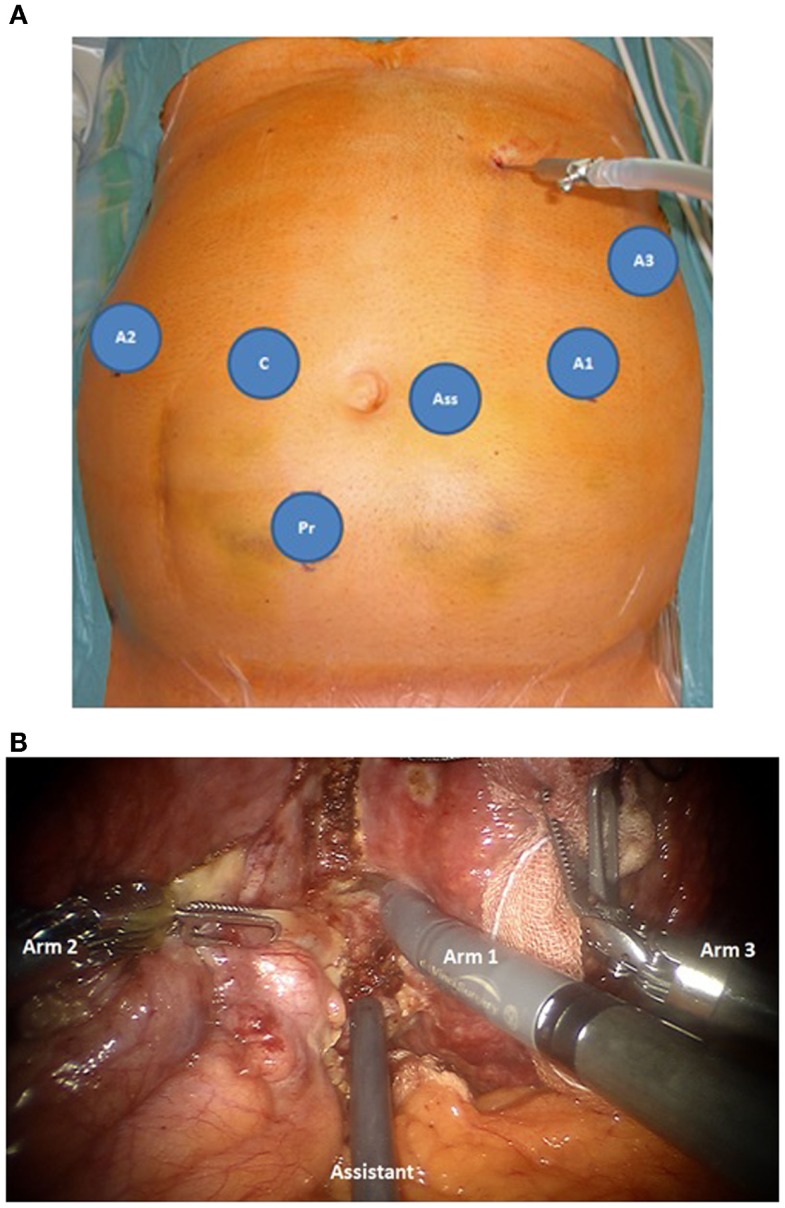
**(A)** Trocar placement for robotic based minimally invasive liver surgery, robotic trocars for arm 1-3 (A1–A3), 10 mm laparoscopic trocars for the camera (C), the surgical assistance (Ass) and if necessary pringle maneuver (Pr). **(B)** Intraopeartive use of the robotic arms. Arm 1: monopolar scissors, arm 2 bipolar forceps, both for tissue preparation, arm 3: liver exposure. The liver tissue is protected by using a sponge. Via the assistant port a laporoscopic succor is inserted to reduce smoke exposure.

**Figure 3:**
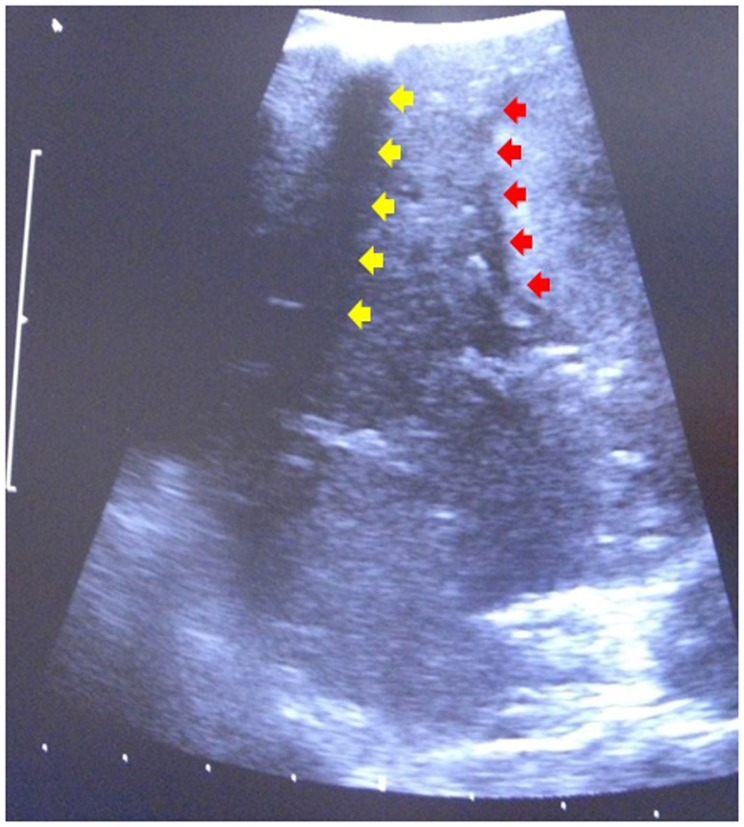
**Laparoscopic ultrasound to identify resection margin**. Red arrows indicate the tumor margin, yellow arrow indicates a shadow induced by an incision of the liver capsular.

**Figure 4:**
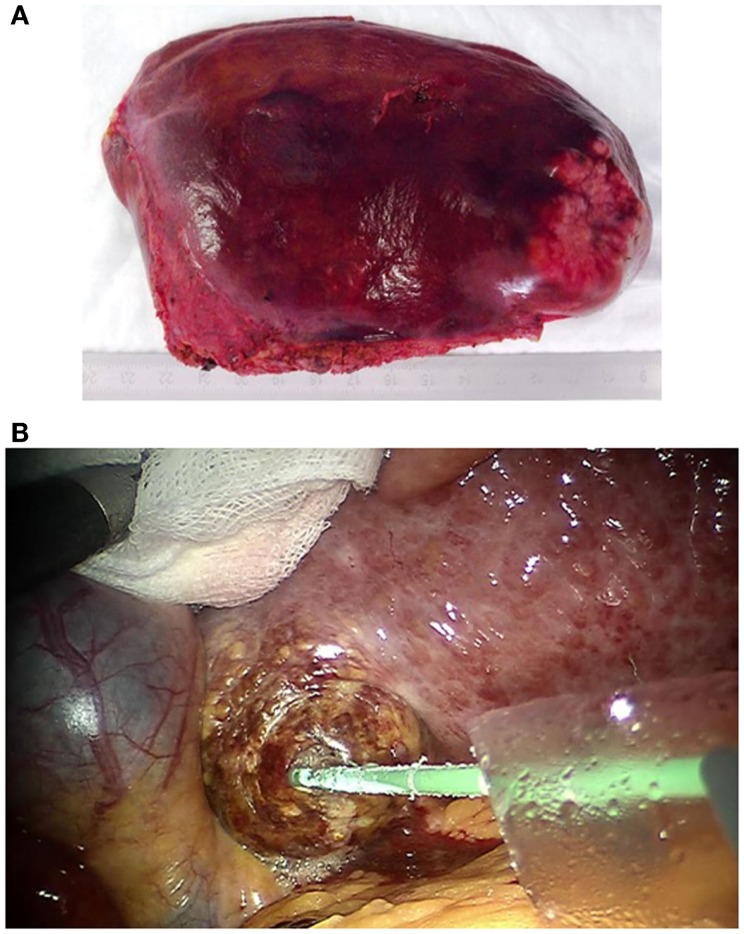
**(A)** Robotic resected and via pfannenstiel incision removed left lateral liver containing rectal cancer liver metastasis. **(B)** Robotic guided HCC microwave ablation. The needle is placed via a 10 mm laparoscopic trocar. A sponge is used to protect the liver tissue during the exposure.

For robotic-based liver resection, we used the Da Vinci Si System (Intuitive Surgical, Inc., CA, USA). Patients were placed in a reverse Trendelenburg position. The screen was located at the head end of the patient. The robot cart moved over the right shoulder. The anesthesia was positioned at the left, the assisting nurse at the right and the assisting surgeon between the legs of the patient (Figure 1). A pneumoperitoneum was achieved puncturing the abdomen with a veress needle in the left upper abdomen. In case of adhesions resulting from prior surgery pneumoperitoneum and explorative laparoscopy can be initiated via a 5 mm port in the same position. The camera of the robot was positioned right above the umbilicus via a 10-mm trocar. If necessary, a 10-mm trocar can be placed below the umbilicus for pringle maneuver. To the left side of the umbilicus, a 10-mm trocar for laparoscopic assistance was inserted. The robotic arm 1 was positioned in the left middle abdomen, the robotic arm 2 in the right upper, and the robotic arm 3 in the left upper abdomen (Figure 2A). Arm 3 was mainly used for liver exposure while via arm 1 and 2 a bipolar forceps and monopolar scissors were placed for tissue preparation (Figure 2B). Liver parenchymal dissection was carried out with harmonic scalpel, which can be inserted via each robotic arm needed. Via the assistant port a laparoscopic succor was placed to reduce smoke exposure or to maintain visualization in case of bleeding. Vessel clipping was performed by using robotic clips, which we prefer for bigger vessels or via the assistant port with laparoscopic clips. The portal or liver vein can be divided by a stapler via the assistant port. Usually, we started the preparation by defining the resection margins with laparoscopic ultrasound. After incising the liver capsular, a shadow can be identified, this shadow marks the distance between liver lesion and resection margin (Figure 3). In a next step, and if necessary, the liver side containing the tumor was mobilized and extrahepatic vessels were dissected. Then, the parenchyma dissection started. Intrahepatic vessels and bile ducts were clipped or stitched. The resected specimens were inserted in a bag and removed via a pfannenstiel incision or in case of prior open surgery using preexisting scars (Figure 4A). Usually, a drain was placed which was removed on postoperative day 2. The fascia was stitched and skin incisions were glued.

In one case (case 6, Table 1), only liver ablation could be performed resulting from unexpected intrahepatic findings. In this patient, microwave ablation was used. The needles were placed through the trocars or percutaneously guided by ultrasound. The liver tumors were exposed using the robotic arms to protect neighboring tissue (Figure 4B).

## Results

### Perioperative variables

Six patients had undergone prior surgery and suffered from adhesions, which were less in the minimally invasive pre-operated cases. In six patients, a left lateral resection and in two cases a single segmental resection were performed. In two cases, the gallbladder was removed for stones or tumor adhesion. Initially, in one patient (case 6, Table 1) left hemihepatectomy was considered but for reasons of unexpected intraoperative identified liver zhirrosis, which prohibited major resection, only microwave ablation of HCC in segments II/III and IV were performed after liver biopsy. The mean operation time was 312 min (range 115–458 min). A 60 min preoperative time for pre-setting the robot and 30 min postoperative time for re-setting the robotic system must be considered. The mean estimated blood loss was 251 ml (range 10–650 ml). No red blood cell substitution was necessary. The mean hospital stay of the patients was 6 days (range 3–10 days). The need for minimum hospital stay to achieve complete reimbursement was respected if possible.

### Histopathology of the resected specimens

In histopathology, in one case CCC, in three cases HCC, and in four cases CRC were confirmed. The mean weight of the resected liver specimens was 182 g (range 62–260). In all resected cases, R0 status was confirmed by histopathology. The men resection margin was 0.6 cm (range 0.1–1.5 cm). Five percent hepatic steatosis was identified in two patients, 5–10% in one patient, and >20% in two patients. Severe liver fibrosis (Ishak 4–6) was present in two cases and less liver fibrosis (Ishak 1–2) in three patients. Steatosis and severe fibrosis was mainly present in patients with primary liver malignancies.

### Morbidity and mortality

Postoperative morbidity (≤30 days postoperative) occurred in one patient. One patient (patient 7, Table 1) developed a small intestine fistula to the camera port on postoperative day 5. The fistula healed spontaneously under conservative treatment and the patient was discharged on postoperative day 10. No bile leaks occurred. Morbidity did not correlate with hepatic steatosis or fibrosis. No postoperative mortality occurred.

### Follow up

During a mean follow up of 12 months (range 1–21 months), two patients developed tumor recurrence. One patient (patient 1, Table 1) developed CCC recurrence in liver segments IV and VI/VII 8 months after liver resection. The initial resection margin in this case was 1.5 cm. This patient is currently under chemotherapy. One patient (patient 7, Table 1) developed tumor recurrence in liver segments V and VI 4 weeks after liver operation. In these segments, no tumor was identified prior to surgery. The lesions were ablated interventionally. No patient died during follow up.

## Discussion

Our experience demonstrates that robotic-based minimally invasive surgery is feasible for primary and secondary liver malignancies. Complete and oncological adequate removal of the tumors is possible. Liver parenchymal disorders like steatosis or fibrosis are no limitations for this technique but correlate with increased operation time. In these cases, the identification for intrahepatic structures is more time consuming. Even adhesions after prior surgery are no contraindications for robotic-based procedures. In case of expected relevant adhesions, the situation can be clarified by explorative laparoscopy via a 5-mm trocar in the left upper abdomen after inducing the pneumoperitoneum. If necessary, laparoscopic mobilization of adherent tissue to the ventral abdomen can be performed. Still operation time is longer and blood loss slightly higher than in open procedures. But even in series of minor resections beyond 25 cases, a mean operation time of 4 h for conventional laparoscopic liver surgery was described (7). Comparing larger international series, laparoscopic resections last usually longer than comparable open procedures and in a compared analysis between laparoscopic vs. robotic resections the time for the robotic procedures was prolonged (2, 8). In this series, a mean operation time of 253 min with the robot and a room covering time of 342 min was described (8). This observation was persisting even in minor resections within the study and is in concordance with our experience that pre-setting and re-setting of the robot covers around 90 min even in an experienced team. Generally, a mean blood loss of 250 ml for laparoscopic liver resections is described, which was within our range (2). But for minor resections it should be much less. Comparing minor robotic vs. laparoscopic liver resections, a mean estimated blood loss of 285 vs. 50 ml was recently identified (8). These observations elucidate the demand of special operation techniques for robotic procedures. Conventional laparoscopic techniques cannot be easily transferred to the robot but must be adapted for its special needs. But during the initiation phase of minimally invasive liver operation the robotic-based surgery gives the surgeon a more comfortable and safe feeling. The outstanding visualization, three easy handling arms with precise instruments and one additional assistant instrument in a stable operation field produce a straight forward and controllable scenario for beginners. The liver can be exposed very well, bleeding can be controlled and stitching using an endowrist is much more easily compared to conventional laparoscopy. Even intrahepatic structures can be well exposed by preparing the tissue with the monopolar scissors. Nevertheless, the perspective of the abdomen is quite different compared to open procedures. For this reason, it is indispensable to be familiar with the liver anatomy. The resection margins can be identified very well by laparoscopic ultrasound. But the resection line must be respected during the whole operation, which might be challenging sometimes because there is no haptic feedback and the tumor cannot be palpated intraoperative. Furthermore, we demonstrated that changing intraoperative strategies such as minimally invasive guided tumor ablation is possible with the robot.

The robot really adds helpful tools for minimally invasive liver resections. But it cannot show its real benefits in minimally invasive segmental resections, which can be carried out even by conventional laparoscopy. These operations are good for starting these procedures and overcoming a learning curve. The highly innovative robotic technique is available and it will stay. The real benefits of robotic based minimally invasive liver resections should be expected in major procedures beyond minor resections with the currently available technology. It remains the surgeons’ preference to identify adequate candidates and indications.

## Ethical Guideline Statement

Informed consent was obtained from all patients and the study was carried out in accordance with institutional and national guidelines and regulations.

## Conflict of Interest Statement

The authors declare that the research was conducted in the absence of any commercial or financial relationships that could be construed as a potential conflict of interest.
